# Bilateral Superior Altitudinal Hemianopsia with Macular Involvement Confirmed by Multifocal Visual Evoked Potential Testing

**DOI:** 10.7759/cureus.4149

**Published:** 2019-02-27

**Authors:** Fabliha Anbar, Valerie Lerebours, Saad Shaikh

**Affiliations:** 1 Medical Education and Simulation, University of Central Florida College of Medicine, Orlando, USA; 2 Ophthalmology, Orlando Veterans Affairs Medical Center, Orlando, USA; 3 Ophthalmology, University of Central Florida College of Medicine, Orlando, USA

**Keywords:** bsah, hemianopsia, pituitary microadenoma, levothyroxine, bilateral superior altitudinal hemianopsia

## Abstract

We report a case of bilateral superior altitudinal hemianopsia (BSAH) secondary to pituitary microadenoma related inferior optic chiasm damage. A 69-year-old-female developed a BSAH with macular involvement that was initially considered as malingering due to the obscurity of this symptom. The patient presents with multiple risk factors for ischemic disease to the ocular and occipital vessels, persistent migraine, hypothyroidism, and a stable pituitary microadenoma, yet no evidence of tissue ischemia or infarction was noted on imaging that could account for her visual field defects. A prior history of pituitary microadenoma is presumed to be the etiologic cause although the lesion had regressed by the time of presentation.

## Introduction

Visual defects can be caused by a multitude of cerebral, ocular, and optic pathway lesions with the pattern having localization value. Of these defects, bilateral altitudinal hemianopsias with macular (or central visual) involvement are rare. The etiologies for such conditions include bilateral pre-chiasmal lesions such as ischemic optic neuropathy [1-2], pituitary lesions [3-4], and cerebral vascular infarction involving the occipital lobe [5-14]. Herein we report on a patient with bilateral superior altitudinal hemianopsia (BSAH) without macular sparing and, to our knowledge, the first such case supported by multifocal visual evoked potential confirmation (mfVEP) of the field defects.

## Case presentation

**Figure 1:**
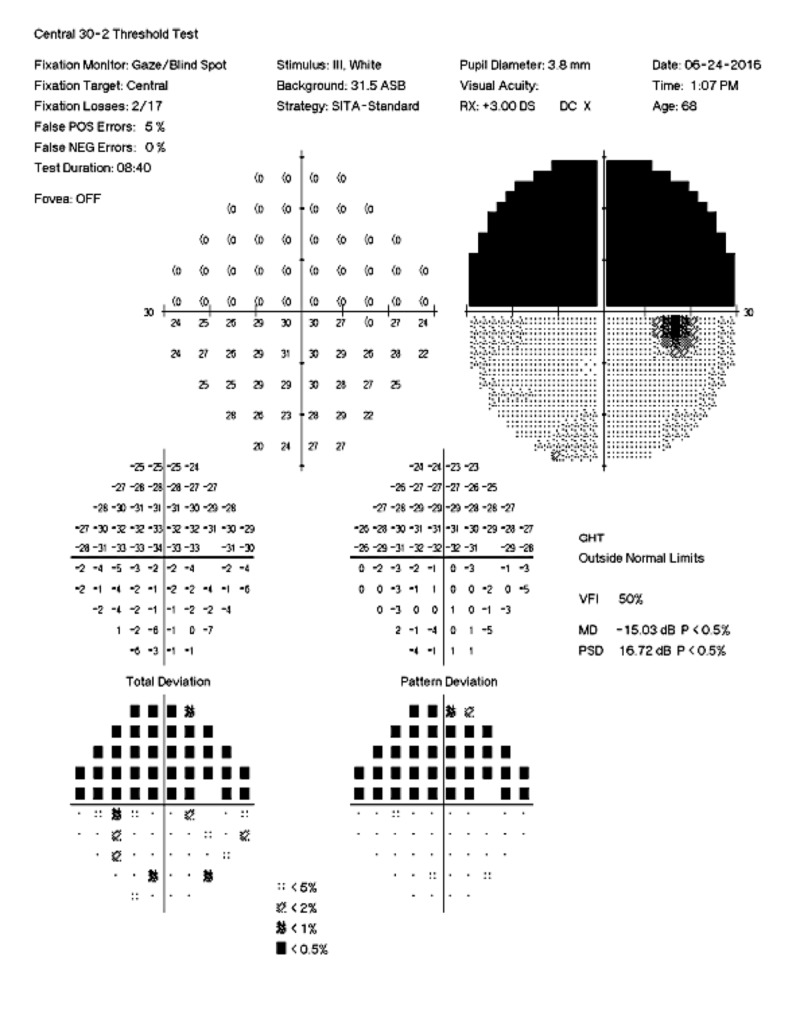
Left Visual Field Automated 30-2 visual field demonstrating complete superior hemifield defect with macular involvement in the left eye.

**Figure 2:**
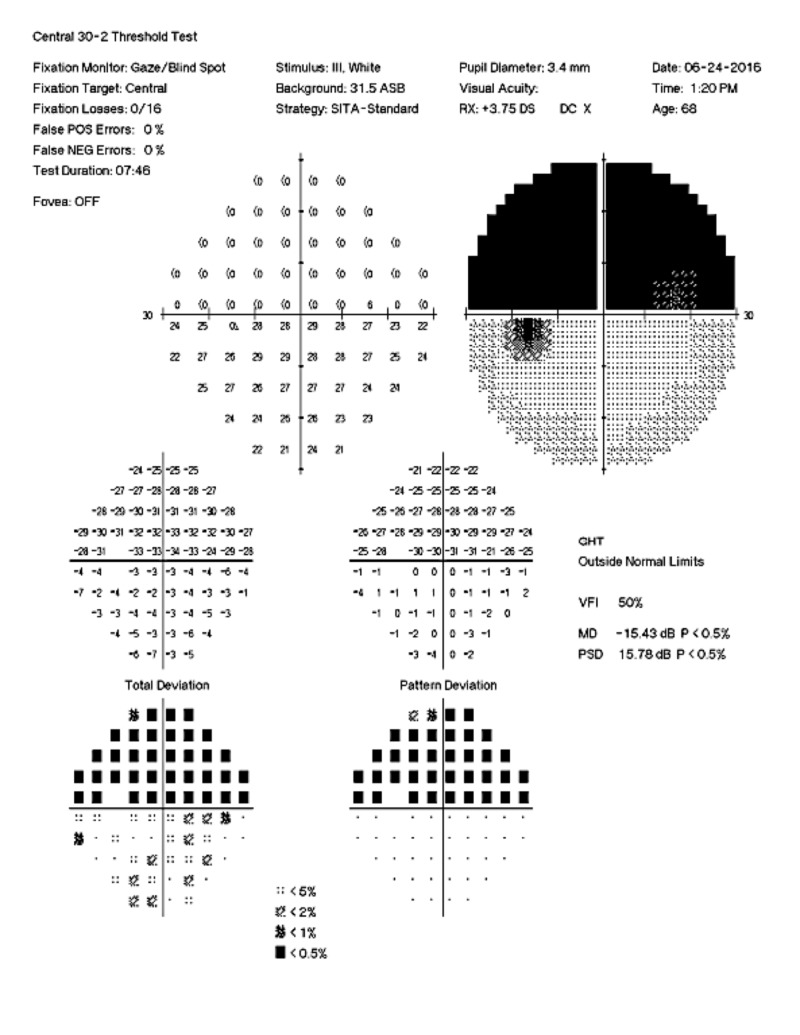
Right Eye Visual Field Automated 30-2 visual field demonstrating complete superior hemifield defect with macular involvement in the right eye.

A 69-year-old female was referred to our visual electrophysiology clinic. The referring optometrist was concerned that the nature of her visual field defects, specifically a bilateral altitudinal hemianopsia with macular involvement, suggested a malingering disorder (Figures 1-2). The visual field changes were first noted 10 years prior, and magnetic resonance imaging (MRI) at the time revealed a pituitary microadenoma not affecting the optic chiasm. Yearly MRI examinations of the brain along with her visual fields remained stable without evidence of growth of the pituitary lesion. Her medical history was also significant for hyperlipidemia, hypertension, mitral valve prolapse, hypothyroidism treated with levothyroxine, and a 20-year history of migraine headaches without visual aura, treated with botulinum toxin injections and oral eletriptan hydrobromide. Her family history was notable for migraines in her mother and sister, the latter also having had a ruptured cerebral aneurysm.

**Figure 3:**
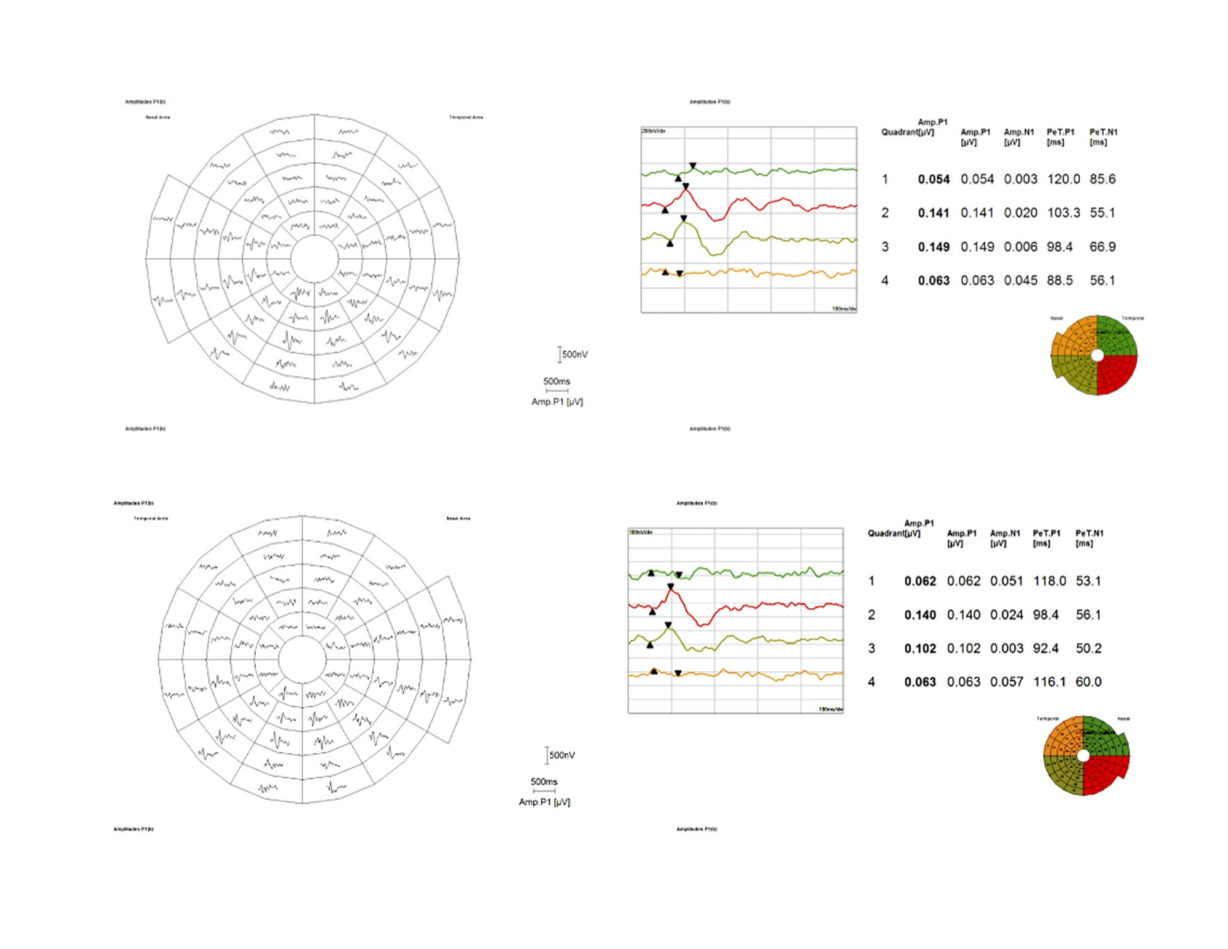
Multifocal Visual Evoked Potential Multifocal visual field (best of responses) of the right (top) and left (bottom) eye demonstrating complete superior visual field loss involving the macula as well. The image at the top is of the right eye, the bottom image is of the left eye.

Her physical examination was unremarkable. Ophthalmic examination revealed visual acuity of 20/20 in each eye. The anterior segments were normal. The cup to disc ratios were 0.3 with healthy appearing nerves bilaterally. Dilated funduscopic examination was normal in both eyes except for lattice degeneration in the inferior periphery of the right eye. Confrontational visual fields were absent superior to the midline. Optical coherence tomography testing of the optic nerve head and macula were normal. Color vision testing was normal in both eyes. A mfVEP was performed and confirmed bilateral superior altitudinal visual deficits with macular involvement (Figure 3). Repeated MRI and magnetic resonance angiogram (MRA) testing of the brain and orbit revealed a hypoenhancing lesion of the right side of the sella turcica, consistent with a pituitary microadenoma, measuring 7.8 x 3.6 mm. This was slightly smaller than the previous examination one year before.

## Discussion

We present a patient with a BSAH with macular involvement confirmed by mfVEP testing. Such lesions are exceedingly rare due to the complex sequence of vascular lesions required to produce such defects [13]. The pathogenetic mechanism for reported lesions include bilateral pre-chiasmal lesions, neuropathy [1-2], pituitary lesions that involve only the inferior optic pathway fibers [3-4], bilateral occipital radiation lesions [15], and occipital lesions that respect the calcarine fissure bilaterally and involve the dual vascular supply to the occipital lobe provided by the posterior and middle cerebral [5-14]. One case has been reported of BSAH in a 61-year-old man who was found to have tentorial herniation secondary to a subdural hematoma. His MRI showed bilateral infarction of the inferior tip of the medial occipital lobe. The patient’s MRI results supported his visual field findings [13].

The etiology of the lesion in our patient remains unclear. Both MRI and MRA revealed normal cerebral anatomic and vascular findings thus excluding an occipital lesion. Funduscopic findings and ancillary retinal and optic nerve testing revealed normal anatomy making a pre-chiasmal lesion unlikely as well. Our patient reported a long-standing history of migraines controlled by medications. One reported case of bilateral altitudinal visual field with macular involvement lasting four months was found in a 25-year-old patient who had migraines with aura. The mechanism was attributed to decreased cerebral blood flow without infarction as a byproduct of cortical spreading depression (CSD). CSD is considered to cause aura in migraines; however, our patient had migraines without aura [1]. In addition, it is highly unlikely our patient’s migraine related visual field defects would have persisted over 10 years without further evaluation.

One plausible etiology for this patient’s BSAH with macular involvement is the patient’s history of a pituitary microadenoma. Injury to the optic chiasm is theorized to be the only single point lesion that can have this case presentation [4]. Compressive or infiltrative lesions in the intrasellar and suprasellar regions are considered one of the most common causes of bilateral superior or inferior anopia [3]. Despite the longitudinal MRI findings showing no compression of the optic chiasm, and no enlargement of the pituitary microadenoma, damage to the optic chiasm may have been caused by an enlargement that was not present from the point of her first MRI onwards. Primary hypothyroidism had caused a pituitary microadenoma with suprasellar extension in a previously reported patient, and long-term treatment with levothyroxine was reported to cause regression [16]. Our patient, similarly, was on levothyroxine therapy for hypothyroidism.

## Conclusions

Of note, this patient was initially referred to us under the presumption of malingering. Unlike conventional visual field testing, which is patient dependent and prone to various errors, mfVEP is an objective measure of the patient’s electrical response at the occipital cortex when stimulated by an on/off checkerboard pattern stimulating the patient’s visual fields. A topographical correlation of the perceived visual field is mapped to the activity at the occipital cortex. In our patient, mfVEP unequivocally confirmed that the patient did have a BSAH with macular involvement. Medical providers should be aware of this unique visual defect and the role of mfVEP testing in the testing and diagnosis of presenting patients.
